# MRI signal changes and their association with intervertebral disc disease in canine vertebral endplates

**DOI:** 10.1186/s13620-019-0148-2

**Published:** 2019-10-31

**Authors:** Emma Deards, Dylan N. Clements, Tobias Schwarz

**Affiliations:** The Royal (Dick) School of Veterinary Studies and The Roslin Institute, The University of Edinburgh, Roslin, Midlothian, EH25 9RG Scotland, UK

**Keywords:** Vertebral endplate change, Dog, Vertebral imaging, Hyperintense, MRI

## Abstract

**Background:**

This study was undertaken to determine the relationship between intervertebral endplate changes and intervertebral disc disease. This study was designed as a cross-sectional, observational study. Two hundred thirteen canine MRI scans performed between 2007 and 2014 were retrieved from a digital image archive. Scans which included any sagittal sections of the vertebral column from C1 to S1 were assessed for morphological changes to the vertebral endplate.

**Results:**

There was found to be a significant association between vertebral endplate changes and intervertebral disc disease of the adjacent disc (*P* = 0.021). There was not found to be any significant association between dogs having vertebral endplate changes and having intervertebral disc disease (*P* = 0.38). There was found to be a highly significant association between discs with vertebral endplate changes on both associated vertebrae (bilateral) and having intervertebral disc disease (*P* = < 0.0001).

**Conclusions:**

The presence of endplate changes should alert the observer to closely examine the disc, as intervertebral disc disease is mildly more likely to occur adjacent to these changes.

## Background

Signal intensity changes of vertebral endplates are a common imaging finding in canine MRI scans, and have been associated with intervertebral disc disease [1]. The causal relationship between intervertebral disc disease and endplate changes is not yet fully understood, and therefore one cannot know whether endplate changes precede intervertebral disc disease or vice versa.

Canine intervertebral disc disease is defined as structural failure of the intervertebral disc associated with abnormal or accelerated changes seen in aging. Hansen type I intervertebral disc disease is characterized by chondroid degeneration of the nucleus pulposus, whereas Hansen type II intervertebral disc disease is characterized by fibrinoid degeneration of the nucleus pulposus [7]. Type I intervertebral disc disease is seen more commonly in chondrodystrophoid breeds in which there is acute complete rupture of the annulus and extrusion of the nucleus pulposus into the vertebral canal, and has a genetic component to its etiology. Type II intervertebral disc disease occurs as a feature of aging and chronic protrusion of the annulus fibrosus. It is seen more commonly in large breed dogs [2].

To date, there has only been one study on the MRI patterns of vertebral endplate changes in dogs, which found that endplate changes were most commonly associated with discospondylitis and fatty infiltration, but also with reactive endplates, osteochondrosis, and intervertebral disc herniation [5]. In that study, however, the direct association between intervertebral disc disease and signal intensity changes was not described. The purpose of this study was to determine whether there was an association between vertebral endplate changes and intervertebral disc disease of adjacent discs.

## Results

**Fig. 1:**
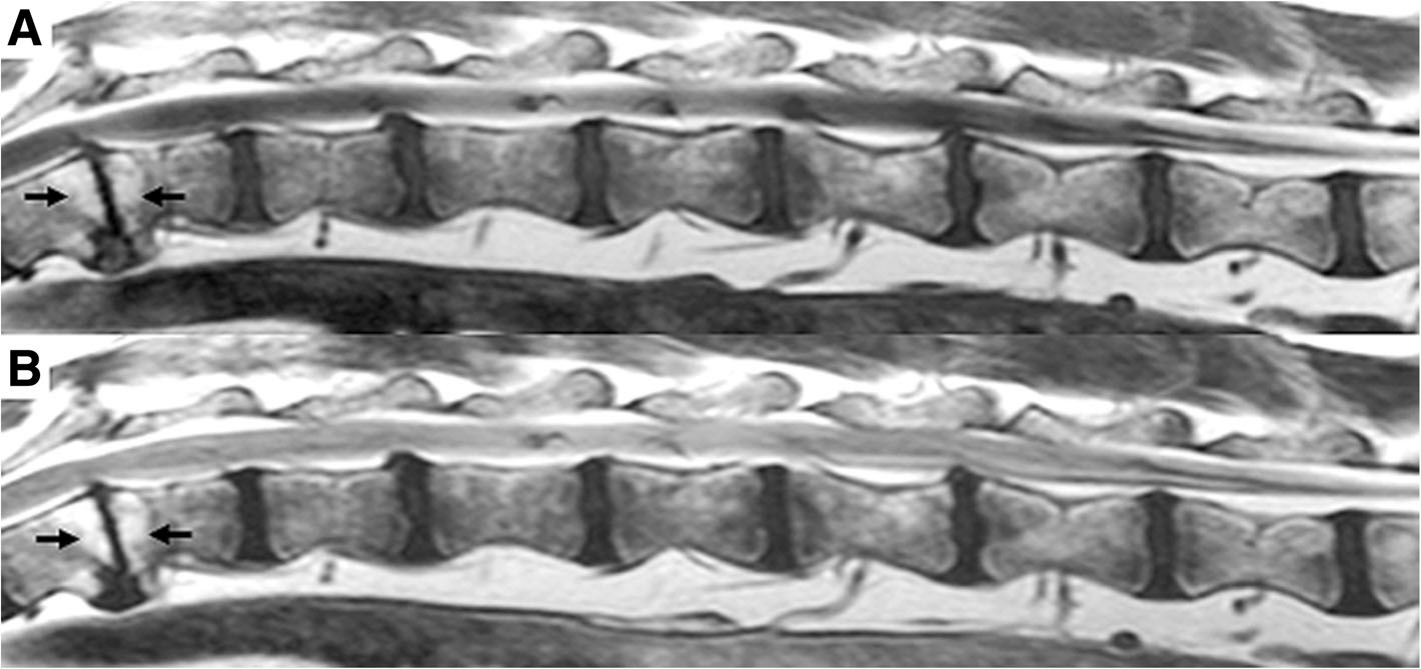
Sagittal image of the vertebral column of a Welsh springer spaniel (male, 3 years old) from T12-L7, showing hyperintense changes (arrows) along the vertebral endplates of intervertebral disc space T12–13 in **a** a T1 weighted image and **b** a T2 weighted image

Two hundred and one dogs with MRI scans met the inclusion criteria for this study. Thirty-six dogs (16%) had at least one type of vertebral endplate change (Tables 1, 2, and 3, and Fig. 1). Of the 36 dogs with vertebral endplate changes present 14 (39%) had only one vertebral endplate change while 22 (61%) had multiple vertebral endplate changes. In the match case-control analysis we evaluated 72 dogs and 1241 intervertebral disc sites. Intervertebral discs that were adjacent to vertebral endplate changes were more likely to be diseased than intervertebral discs that were not (OR = 1.71, CI = 1.08–2.71, *P* = 0.021, Table 4). 21.3% of total vertebral endplate changes seen had changes on both associated vertebral endplates (bilateral), whereas 78.6% had changes on only one associated vertebral endplate.

**Table 1:** Breeds of dogs examined

Breed type	Change group	Control group
American Cocker Spaniel	0 (0.0%)	1 (2.77%)
Beagle	0 (0.0%)	1 (2.77%)
Border Collie	0 (0.0%)	1 (2.77%)
Boxer	2 (5.55%)	2 (5.55%)
Cavalier King Charles Spaniel	1 (2.77%)	1 (2.77%)
Cocker Spaniel	7 (19.44%)	6 (16.66%)
Collie Cross	2 (5.55%)	1 (2.77%)
Cross Breed	0 (0.0%)	1 (2.77%)
Greyhound	2 (5.55%)	2 (5.55%)
Hungarian Vizsla	1 (2.77%)	1 (2.77%)
Jack Russel Terrier	2 (5.55%)	2 (5.55%)
Labrador Retriever	3 (8.33%)	4 (11.11%)
Lhasa Apso	0 (0.0%)	1 (2.77%)
Lurcher	1 (2.77%)	1 (2.77%)
Mini Dachshund	2 (5.55%)	0 (0.0%)
Mini Schnauzer	1 (2.77%)	0 (0.0%)
Pug	1 (2.77%)	1 (2.77%)
Schnauzer	0 (0.0%)	1 (2.77%)
Siberian Husky	1 (2.77%)	1 (2.77%)
Springer Spaniel	1 (2.77%)	1 (2.77%)
Staffordshire Bull Terrier	1 (2.77%)	0 (0.0%)
Terrier	2 (5.55%)	0 (0.0%)
Tibetan Terrier	1 (2.77%)	1 (2.77%)
Weimaraner	1 (2.77%)	1 (2.77%)
Welsh Springer Spaniel	1 (2.77%)	0 (0.0%)
West Highland White Terrier	1 (2.77%)	3 (8.33%)
Yorkshire Terrier	2 (5.55%)	2 (5.55%)
Total	36 (100.0%)	36 (100.0%)

**Table 2:** Sex distribution of dogs examined

	Change group	Control group
Neutered Male	10	11
Neutered Female	11	9
Male	14	10
Female	1	6
Total	36	36

**Table 3:** Mean and median ages of dogs examined

	Change group	Control group
Mean	8.5	6.25
Median	9	6

**Table 4:** Number of intervertebral disc disease positive/negative, vertebral endplate change positive/negative intervertebral discs, and associated statistics related to each group criteria

Intervertebral disc sites	Intervertebral disc disease positive	Intervetebral disc disease negative	Total
Vertebral Endplate Change Positive	30	58	88
Vertebral Endplate Change Negative	268	885	1153
Total	298	943	1241
*p*-value	Odds ratio	Confidence interval 95%	
0.021	1.71	1.08–2.71	

Dogs that had vertebral endplate changes present anywhere in their vertebral column, however, were not more likely to have intervertebral disc disease than dogs without vertebral endplate changes (OR = 1.67, CI = 0.52–5.34, *P* = 0.38, Table 5).

**Table 5:** Probability of disease presence relating to presence or absence of vertebral endplate change

Dogs	Intervertebral disc disease positive	Intervertebral disc disease negative	Total
Vertebral Endplate Change Positive Dog	30	6	36
Vertebral Endplate Change Negative Dog	27	9	36
Total	57	15	72
*p*-value	Odds ratio	Confidence interval 95%	
0.38	1.67	0.52–5.34	

Discs that had vertebral endplate changes on both associated vertebrae (bilateral) were significantly more likely to have intervertebral disc disease, but this presentation was less common (OR = 7.12, CI = 2.68–18.91, *P* = < 0.0001, Table 6).

**Table 6:** Probability of disease presence relating to presence of bilateral vertebral endplate changes

	Intervertebral disc disease positive	Intervertebral disc disease negative	Total
Vertebral Endplate Change Positive Discs	12	6	18
Vertebral Endplate Change Negative Discs	286	937	1223
Total	298	943	1241
*p*-value	Odds ratio	Confidence interval 95%	
< 0.0001	6.5525	2.44–17.61	

## Discussion

The results of this study suggest that T1- and T2-hyperintense changes of the canine vertebral endplate are significantly associated with the presence of intervertebral disc disease. A previous study suggested that there was an overlap between signal patterns of reactive changes, discospondylitis, and vertebral osteochondrosis in dogs [3], although neither of these conditions were observed in our study. As intervertebral disc disease is a common incidental finding on MRI evaluation of the canine vertebral column, their role in contributing to vertebral column pathology warrants further consideration. In particular, undertaking a longitudinal re-evaluation of cases where vertebral endplate changes were identified without intervertebral disc disease would be of value, in order to assess whether vertebral endplate changes are precedent of intervertebral disc disease development. In this study, intervertebral disc disease was as common in dogs with vertebral endplate changes as in dogs without them (Table 5.) The majority of the vertebral endplate changes that were noted in the study was hyperintense in both T1WI and T2WI (35 out of 36–97.22%).

Some limitations in this study were the subjectivity involved in locating and typing the vertebral endplate changes, as well as the grading of the intervertebral discs and the treatment of each intervertebral disc in the chi-squared evaluation as an independent unit, although multiple intervertebral discs came from the same animal (and are therefore likely to share a disease status). This was accounted for to a degree by matching vertebral endplate change positive dogs with dogs with a similar signalment, and by comparing dogs and intervertebral discs independently. The small sample size of this study is a limitation in that the average age of these two groups was not matched as well as would have been ideal. The association of age, vertebral endplate changes, and intervertebral disc disease should be the subject of future study. Another limitation is that due to the subjectivity in disc evaluation it would be difficult to replicate the study. Additionally, since the causal relationship between endplate changes and intervertebral disc disease is not understood, we cannot know whether intervertebral disc disease is caused by endplate changes or the changes are caused by intervertebral disc disease. Further research is needed to understand the causal relationship of the disease process. Finally, the length of the vertebral column evaluated was not part of the inclusion criteria for this study. Thus it is possible that some vertebral endplate changes would be missed by virtue of not examining the entire vertebral column in each animal. Assessing MRI images blinded to disease was sometimes impossible in those cases where there was obvious gross pathology present in the scan.

## Conclusions

Vertebral endplate changes were significantly associated with the presence of intervertebral disc disease of the adjacent disc. Dogs with vertebral endplate changes were not, however, more likely than vertebral endplate change-negative dogs to have intervertebral disc disease. Larger cohort studies of canine vertebral endplate changes are required to determine if they have value in diagnosing, prognosticating, and treating canine vertebral column disease.

## Methods

This study was designed as cross-sectional, observational study. The digital diagnostic imaging archive of the Royal (Dick) School of Veterinary Studies was reviewed for canine vertebral column MRI scans performed between 2007 and 2014. Inclusion criteria were MRI scans that were: of dogs; that featured a sagittal plane of any portion of the vertebral column from C1-S1; and which included both T1 and T2 weighted images. The inclusion and exclusion criteria were set by a board-certified veterinary radiologist (TS) and medical record findings were recorded by a licensed veterinarian (ED). Exclusion criteria were those of non-canine subjects, any scans that did not have a sagittal plane of any portion of the vertebral column from C1-S1, and those which did not include both T1 and T2 weighted images.

The scans were reviewed by TS and ED independently for presence of intervertebral disc disease and endplate changes. Consensus opinions were reached for all findings. The signalment (age, breed, sex), reason for scan, and duration of problem were recorded. Each spinal column was assessed for vertebral endplate changes and their locations (if present) were recorded. Each MRI T1WI and T2WI was evaluated for signal intensity changes at or around the end plates. Both observers were blinded to the case history, signalment, and clinical findings at the time of the MRI. Vertebral endplate changes were determined to be present based on subjective presence or absence of hyperintense or hypointense changes at or along the vertebral endplate.

Dogs with vertebral endplate changes were matched to a subpopulation of dogs that did not have vertebral endplate changes present as a control group. Only these two groups of dogs were included in the analysis. The dogs were matched based on their signalment as closely as possible to the vertebral endplate change-positive dogs in order to best emulate the disease positive group, but examinations were conducted blind to signalment. Breed was weighted more heavily than age or sex in matching. The intervertebral discs of the vertebral endplate change-positive and vertebral endplate change-negative dogs were identified as either undiseased or diseased based their subjective appearance as follows: those intervertebral discs that appeared normal and those slightly degenerated with mild or partial loss of hyperintensity of the intervertebral discs in T2 weighted scans were identified as undiseased; intervertebral discs that were very degenerated with complete loss of hyperintensity of the intervertebral discs in T2 weighted scans, and those that were very degenerated with complete loss of hyperintensity of the intervertebral discs in T2 weighted scans with evidence of intervertebral disc herniation, extrusion, or disc space collapse, were both considered to be diseased for the purposes of this study [6]. Dogs with vertebral endplate changes present on their scans were noted and re-evaluated 4 weeks later, with the same reviewer re-evaluating each intervertebral disc without knowledge of its previous assessment, to ensure consistency. There was complete agreement with the initial findings. Location of the vertebral endplate change along the vertebral column (C2-S1) was noted and recorded. In cases where both associated endplates (bilateral) exhibited a change, each change was recorded separately. Bilateral pairs of endplate changes were noted and calculated to produce the data in Table 6.

### Statistical analysis

Only dogs with vertebral endplate changes and matched control dogs were used for analysis. Dogs with at least one vertebral endplate change were categorized as vertebral endplate change-positive. The data for all included scans (positive and control) were analysed with commercial software by ED [4]. All intervertebral discs for all included dogs were categorized into vertebral endplate change positive/negative, and intervertebral disc disease positive/negative. Chi-square tests and odds ratios were calculated to discern and quantify any associations with the signal changes and disease. The data were evaluated twice to identify the associations between intervertebral disc disease and vertebral endplate changes. First, intervertebral discs were categorized as disease positive and adjacent to a vertebral endplate change, disease negative and adjacent to a vertebral endplate change, disease positive and not adjacent to a vertebral endplate change, and disease negative and not adjacent to a vertebral endplate change. Second, each dog was categorized as disease positive and vertebral endplate change-positive, disease negative and vertebral endplate change positive, disease positive and vertebral endplate change negative, and disease negative and vertebral endplate change negative. The first analysis was intended to assess whether being adjacent to a vertebral endplate change increased the risk of intervertebral disc disease. The second was intended to assess whether a dog with any vertebral endplate changes was more likely to have intervertebral disc disease present than a dog without vertebral endplate changes. The resulting data were evaluated using chi-square test and odds ratios. A *P*-value < 0.05 was determined as demonstrating significance.

## Data Availability

The dataset analysed during this study is available from the corresponding author on reasonable request.
